# Oil palm in the 2020s and beyond: challenges and solutions

**DOI:** 10.1186/s43170-021-00058-3

**Published:** 2021-10-11

**Authors:** Denis J. Murphy, Kirstie Goggin, R. Russell M. Paterson

**Affiliations:** 1 School of Applied Sciences, University of South Wales, Pontypridd, CF37 4AT UK; 2 School of Pharmacy and Pharmaceutical Sciences, University of Cardiff, CF10 3NB Cardiff, UK; 3 CEB-Centre of Biological Engineering, Gualtar Campus, University of Minho, 4710-057 Braga, Portugal; 4 Department of Plant Protection, Faculty of Agriculture, Universiti Putra Malaysia, 43400 UPM Serdang, Selangor D.E. Malaysia

**Keywords:** Oil palm, Breeding, Sustainability, Diseases, Basal stem rot, *Phytophthora*, Climate change, Modelling

## Abstract

**Background:**

Oil palm, *Elaeis guineensis*, is by far the most important global oil crop, supplying about 40% of all traded vegetable oil. Palm oils are key dietary components consumed daily by over three billion people, mostly in Asia, and also have a wide range of important non-food uses including in cleansing and sanitizing products.

**Main body:**

Oil palm is a perennial crop with a > 25-year life cycle and an exceptionally low land footprint compared to annual oilseed crops. Oil palm crops globally produce an annual 81 million tonnes (Mt) of oil from about 19 million hectares (Mha). In contrast, the second and third largest vegetable oil crops, soybean and rapeseed, yield a combined 84 Mt oil but occupy over 163 Mha of increasingly scarce arable land. The oil palm crop system faces many challenges in the 2020s. These include increasing incidence of new and existing pests/diseases and a general lack of climatic resilience, especially relating to elevated temperatures and increasingly erratic rainfall patterns, plus downstream issues relating to supply chains and consumer sentiment. This review surveys the oil palm sector in the 2020s and beyond, its major challenges and options for future progress.

**Conclusions:**

Oil palm crop production faces many future challenges, including emerging threats from climate change and pests and diseases. The inevitability of climate change requires more effective international collaboration for its reduction. New breeding and management approaches are providing the promise of improvements, such as much higher yielding varieties, improved oil profiles, enhanced disease resistance, and greater climatic resilience.

## Introduction

The palms, or Arecaceae, are a family of stem-less, tree-like monocot plants that are highly significant to humans and wider biodiversity, especially in the tropics (Cosiaux et al. 2018). The African oil palm, *Elaeis guineensis*, is native to West Africa and in terms of agriculture, it is perhaps the world’s most important palm species. Oil palm fruits are available year-round and have served as semi-wild food resources in traditional societies for > 7000 years. In its regions of origin the oil palm plant has great significance to local people and for wider biodiversity (Cosiaux et al. 2018; Reddy et al. 2019; Okolo et al. 2019). Cultivation of oil palm as a crop was originally an informal process mainly confined to the West/Central African coastal belt between Guinea/Liberia and Northern Angola (Corley and Tinker 2015). Globally, the best production levels are achieved in high rainfall areas in equatorial regions between 7° N and 7° S. During the nineteenth century, oil palm seeds were transported to the Dutch East Indies (modern Indonesia), and to the Malay States (modern Malaysia), as part of colonial ventures to grow newly introduced cash crops in the region. During the twentieth century, more systematic oil palm cultivation on plantations gradually became established in the Malay States. In terms of large-scale commercial production, however, oil palm is a relatively recent crop that only emerged into global prominence later in the twentieth century, with an almost linear rise from 1990 to the early 2000s, followed by a plateau after 2007 (Malaysian Palm Oil Production by Year 2020). This was largely due to government initiatives in the 1970s and 80 s aimed at improving the agriculture and economy of the newly independent nation of Malaysia (Corley and Tinker 2015; Murphy 2014). The later rise of the oil palm industry in Indonesia occurred during the twenty-first century when there was a > 5-fold increase in oil production from 8.3 Mt in 2000 to 43.5 Mt in 2020.

Today, oil palm is crucial to the economies of many countries, especially Indonesia and Malaysia, from which large quantities of its products are exported in the form of oil, meal and other derivatives (Murphy 2019). More widely, oil palm is now cultivated in plantations across the humid tropics of Asia, Africa and the Americas, from where its products are exported to global markets. However, despite its increasing cultivation on three widely separated continents, the vast majority of oil palm is still grown in the two adjacent South East (SE) Asian countries of Indonesia and Malaysia (Table 1) that generate about 85% of the entire global production (Murphy 2014, 2015, 2019; Statista 2020; Goggin and Murphy 2018). The major importing regions, collectively responsible for about 60% of total palm oil imports, are the Indian subcontinent (India, Pakistan, Bangladesh) with about 17 Mt, the EU-27 with 6.5 Mt, and China with 5 Mt (Statisa 2020).

**Table 1:** Major centres of global oil palm cultivation in 2020. Source: Goggin and Murphy (2018)

Rank	Country	Palm oil production	
		Mt	%
1	Indonesia	42.5	58.8
2	Malaysia	18.5	25.6
3	Thailand	2.8	3.9
4	Colombia	1.5	2.1
5	Nigeria	1.0	1.4
	Others	5.9	8.2
	Total	72.3	

There are two contrasting types of oil found in the two principal tissues of palm fruits, namely ‘palm oil’ and ‘palm kernel oil’ (Murphy 2019). Palm oil, extracted from the fleshy mesocarp tissue, is a deep orange-red, semi-solid fluid, whilst palm kernel oil is a white-yellow oil that is extracted mainly from the endosperm tissue of the kernel (seed). These two oils have very different fatty acid compositions (Table 2), which means they are used for different downstream applications in a range of industrial sectors (Goggin and Murphy 2018). In general, the relatively high saturated fat content of palm oil makes it particularly suitable for edible use as a solid vegetable fat (melting point ca. 35 °C). In contrast, palm kernel oil is a less dense product (melting point ca. 24 °C) that is mostly used for non-edible applications (Statisa 2020). A major use of palm kernel oil is as the key functional ingredient in many soaps, detergents and cosmetics. *E. guineensis* plants bear prolific numbers of oil-rich fruit bunches year-round, each containing between 1000–3000 individual fruits (Corley and Tinker 2015). Mesocarp-derived palm oil makes up about 89% the total fruit oil with the remaining 11% being derived from the seed or kernel. Because palm oil and palm kernel oil are extracted from fruits by different mechanical processes and have very different downstream uses, they enter separate supply chains immediately after extraction in mills.

**Table 2:** Principal fatty acid compositions of the nine major globally traded vegetable oils

Crop	% global supply	Principal fatty acids						
		12:0 Lauric	14:0 Myristic	16:0 Palmitic	18:0 Stearic	18:1 Oleic	18:2 Linoleic	18:3 α-Linolenic
Oil palm (mesocarp)	35.5		1	43	4	40	10	0.3
Oil palm (kernel)	4.3	48	16	8	2	15	2.5	
Soybean	27.8			11	4	23	54	8
Rapeseed	13.4			4	2	60	20	10
Sunflower	10.4			7	5	19	68	
Peanut	3.0			12	5	48	30	
Cottonseed	2.5		1	24	2	18	54	0.5
Coconut	1.8	49	17	9	2	6	2	
Olive	1.5			13	2	70	13	0.6

Saturated fatty acids are in blue, monounsaturates in red and polyunsaturates in black. In each case, data reflect average vales from the main commodity varieties and do not reflect specialist niche varieties such as high-erucic rapeseed or high-oleic soybean oils. In 2019–20, the total production of these nine vegetable oils was about 204 Mt. Data from refs 14, 167

In terms of annual production, the global oil palm industry is worth about US$ 60 billion, employing 6 million people directly plus an additional 11 million indirectly (Kadandale et al. 2019). Over 81.1 Mt of palm oils were produced globally in 2019–20, of which 72.3 Mt was mesocarp oil (hereafter referred to as ‘palm oil’) while 8.8 Mt was palm kernel oil (Statisa 2020). It is estimated that palm oil or palm kernel oil are present as ingredients in at least half of the products found in a typical supermarket. At least three billion people rely directly on palm oil as a regular part of their diet, and it is a staple cooking oil commonly used in African and Asian food preparation. As global populations increase, the demand for palm oil is likely to continue to rise. Estimates from various industry sources predict that between 93 and 156 Mt palm oil might be required by 2050 (Frost and Sullivan 2017; Harris et al. 2013; Pirker et al. 2016). However, these estimates do not consider the effect of climate change on production, which is likely to reduce the ability of the sector to meet these demands (Paterson 2020a, b, 2021a, b) as discussed later.

In addition to its edible applications, the oil palm crop provides a wide range of non-food products that also include animal feeds. These feeds are derived from the seeds or kernels, which contain a protein-rich meal residue following oil extraction. Palm kernel meal is an often overlooked product of the crop, but is a useful livestock feedstuff that is exported globally. In 2019, about 7.6 Mt palm kernel meal was exported, almost exclusively (98%) from Indonesia and Malaysia (Indexmundi 2021). In order of importance, the major importing countries (75% of total 2019 imports) are the EU, New Zealand and Japan, where the meal is used in a variety of feed formulations, especially for ruminants such as cattle.

The image of oil palm has been adversely affected by detrimental environmental consequences of its cultivation, especially with respect to deforestation and haze creation (Paterson and Lima 2018). There is also great public concern about the plight of iconic species, and particularly the orangutan, in SE Asia. Some of the particular challenges currently faced by the industry include the following:

- Greatly reduced demand for crop-derived biofuels, especially in Europe.
- Serious production issues related to plantation management, labour shortages, replanting with improved crop varieties, mechanization etc.
- Ongoing environmental and sustainability issues including deforestation, biodiversity loss and GHG emissions due to crop expansion.
- Growing threats arising from climate change, including biotic factors, such as pests and disease, that could impact crop performance in unpredictable ways.
- Increasingly serious supply chain and consumer issues including potential trade barriers and boycotts.

In all cases these issues will require attention by the industry during the rest of this decade and beyond.

## Structure of the oil palm industry

Modern commercial oil palm cultivation began in Malaysia in 1917 (Basiron 2007) and over 88% of palm oil is still produced by Malaysia plus the neighbouring countries, Indonesia and Thailand (Statista 2020). From 2001 to 2016, the expansion of oil palm plantations was particularly marked in this region with a 2.5-fold increase in Malaysia and a 4.2-fold increase in Indonesia (Xu et al. 2020). Over the past decade, oil palm crops have also been grown increasingly outside SE Asia (Murphy 2019), as suitable land in Asia becomes scarce and the changing climate is less conducive to cultivation (Paterson 2021a, b). For example, there is only an estimated 300,000 ha of available land for palm expansion remaining in Malaysia (Villela et al. 2014), with increasing government prohibitions for environmental reasons, on further encroachment onto forest and peatland in Indonesia (Jackson 2019). Continuing increases in global demand over the past five decades have meant that the cultivation of oil palm has been widely regarded by many tropical countries as a method to boost their economies (Arrieta et al. 2007; Ohimain and Izah 2014; Paterson et al. 2015).

In SE Asia, the primary regions for oil palm production in Indonesia are Sumatra and Kalimantan (Paterson et al. 2015; Suryantini and Wilandari 2018), while in Malaysia the peninsula was the historical centre, although considerable expansion has occurred more recently in Sabah and Sarawak. Due their climatic suitability, oil palm cultivation has also spread to other SE Asian countries, especially Thailand and Papua New Guinea, with Myanmar and the Philippines in the initial stages of development where the crop is important to the economies of each of these countries (Corley and Tinker 2015; Suryantini and Wilandari 2018; Pornsuriya et al. 2013; Somnuek et al. 2016; Woods 2015). Due to its profitability, there are also significant emerging oil palm industries in much of tropical Africa with Nigeria, Ghana, Ivory Coast, Cameroon, Sierra Leon, Benin, Angola, and DRC as the main producers (in that order) (Paterson 2021a). However, in most cases African oil palm crops are mainly used for local consumption, with Cameroon and Ivory Coast as the only major palm oil exporters (Corley and Tinker 2015). Nigeria is the fifth highest producer globally, with an annual 1·0 Mt, although this is dwarfed by Indonesia with 42.5 Mt and Malaysia with 18.5 Mt (Statista 2020).

In the Americas, the first oil palm plantations were established in Honduras and Costa Rica and currently the largest industries are in Colombia and Ecuador, although Brazil is also expanding its production (Corley and Tinker 2015; Murphy 2019; Nahum et al. 2020). South and Central America are considered as favourable areas for oil palm development due to their theoretical ability to produce palm oil. There is well over 1.5 Mha of planted oil palm in Latin America with Brazil having the largest future potential, although currently the leading producer is Colombia with an annual 1.5 Mt. Although the environmental consequences of increasing oil palm cultivation require careful consideration (Murphy 2015; Paterson 2020a, b, 2021a), these countries could potentially increase their market share in a sustainable manner, for example by only converting land currently used for pasture or illegal coca cultivation. This will be important as land in Malaysia and Indonesia becomes less available (Paterson and Lima 2018). However, there are also important climate change constraints for a truly sustainable future industry in the Americas, Africa, and SE Asia (Paterson 2021a, b, c, d; Indexmundi 2021; Paterson et al. 2015, 2017).

A recent study shows the global distribution of smallholder and industrial plantations at high resolution (Descals et al. 2020). Smallholders account for 30 to 40% of global land palm oil cultivation (Hambloch 2018; Euler et al. 2016). In SE Asia there are more than three million smallholders, nearly all of whom cultivate individual family-owned and managed plots of less than 50 ha and often as little as 1–2 ha. In Indonesia, which is the largest oil palm producing country, smallholder plots account for 40% of the total crop area, although they only produce 30% of total national output (Euler et al. 2017). However, although the larger commercial plantations tend to be more efficient in terms of oil yield and overall economics, smallholder units serve important social roles in providing income and employment to rural populations (Murphy 2015; Euler et al. 2016). Smallholder units are also more likely to supply palm oil for local consumption rather than for export. This is particularly true for parts of Indonesia and Africa where the crops can be regarded as key elements in local food security and economic wellbeing (Krishna et al. 2017; Kubitza et al. 2018). Interestingly, there is also evidence that smallholdings can have lower environmental impacts (Lee et al. 2014) and higher biodiversity levels than commercial plantations (Razak et al. 2020).

In contrast, commercial plantations tend to be part of large ventures that are often owned by multinational companies that can extend over tens of thousands of hectares, with the largest totalling about one million ha. In terms of global trade, palm oils from commercial plantations are by far the most important contributors. In some cases, the larger plantation companies also own or control many key downstream elements in palm oil supply chains. These include mills, refineries, shipping operations and the distribution networks to processors and retailers in export destinations.

In summary, oil palm cultivation is still highly concentrated in SE Asia, but the focus of future expansion is likely to be elsewhere in the humid tropics, especially in West Africa and northern regions of South America. Therefore, the oil palm industry is a hybrid of large scale, globally focussed, commercial farming and small scale production of a cash crop, often for local consumption. As discussed below, the industry must manage the effects of environmental factors, such as climate change and increased disease incidence on cropping systems, as well as changing consumer sentiments in export destinations.

## The environmental context

Oil palm is widely considered as a problematic crop. This has been mainly due to the environmental and ecological impacts of some of the land conversions to oil palm plantations over the past two decades, especially in Indonesia. In many cases these have displaced pristine tropical habitats and affected iconic wildlife species, such as orangutan (May-Tobin et al. 2012; Gaveau et al. 2014). The EU is the second largest global importer of palm-based oils and this consumer-led demand has been one of the drivers of the expansion of recent oil palm cultivation. Since 2000, increased global demand for biofuels and other non-food products (mainly from Europe), and for food (mainly from India and China), were the major factors behind the conversion of land in SE Asia to oil palm cultivation. In Indonesia the area of oil palm cultivation more than trebled from 2.5 Mha to over 8 Mha between 2000 and 2014 (Indonesia: Palm oil expansion unaffected by Forest Moratorium 2013). In some cases this has led to significant habitat loss and reductions in biodiversity as complex ecosystems are replaced with simpler species-poor plantation systems, as well as concerns about increased GHG emissions as land is converted to oil palm (Dislich et al. 2017; Meijaard et al. 2020; Carlson et al. 2012; Barcelos et al. 2015).

Several studies have examined the potential impact of land use and climate change on biodiversity in Borneo, where a great deal of oil palm planting has occurred during the past decade (Scriven et al. 2015; Gaveau et al. 2016). Recommendations from these and other studies, include the need to establish nature reserves in upland areas where climate change will be less severe and also to improve connections between reserves and plantations via wildlife corridors (Scriven et al. 2019). One of the most controversial aspects of new palm cultivation in SE Asia is the use of tropical peatland, especially in Borneo. There are several ongoing studies of the impact of peatland conversion in terms of GHG emissions, and other environmental studies have been carried out in association with the major certification scheme that is run by the Roundtable on Sustainable Palm Oil (RSPO). Examples include the following articles: (Gunarso et al. 2013; Chase et al. 2012; Dalal and Shanmugam 2015; Tonks et al. 2017; Cook 2018).

As a result of such studies, RSPO insist that to gain certification, new plantings since November 2005, must not have replaced primary forest or any area required to maintain or enhance one or more High Conservation Values (HCV). An HCV assessment, including stakeholder consultation, must be conducted prior to any conversions and dates of land preparation and commencement must also be recorded. The HCV assessment process requires appropriate training and expertise, and must include consultation with local communities, particularly for identifying social HCVs. Development should actively seek to utilize previously cleared and/or degraded land. Plantation development should not put indirect pressure on forests through the use of all available agricultural land in an area. In order to improve the participation of smallholders, local certification schemes, such as the Malaysian Sustainable Palm Oil (MSPO) and Indonesian Sustainable Palm Oil (ISPO) initiatives, have been set up, although internationally traded palm oils are almost exclusively certified by RSPO.

Several studies suggest that limited oil palm expansion might be possible on already degraded land, without the need to convert mature tropical forests (Jackson 2019; Wicke et al. 2011), and that smallholdings may have lower environmental impacts than commercial plantations (Lee et al. 2014). Despite these caveats, there is considerable pressure for governments to impose much stricter controls, and even outright bans, on the conversion of tropical peatlands and non-degraded forest to oil palm. Although there have been encouraging statements along these lines from politicians in the two major producing countries, these remain largely aspirational at present and more effective action is required.

## Pests and diseases

Oil palm crops are affected by several economically important pests and fungal pathogens, of which several of the most serious diseases will now be considered (Corley and Tinker 2015).

### Fungal diseases

**Fig. 1:**
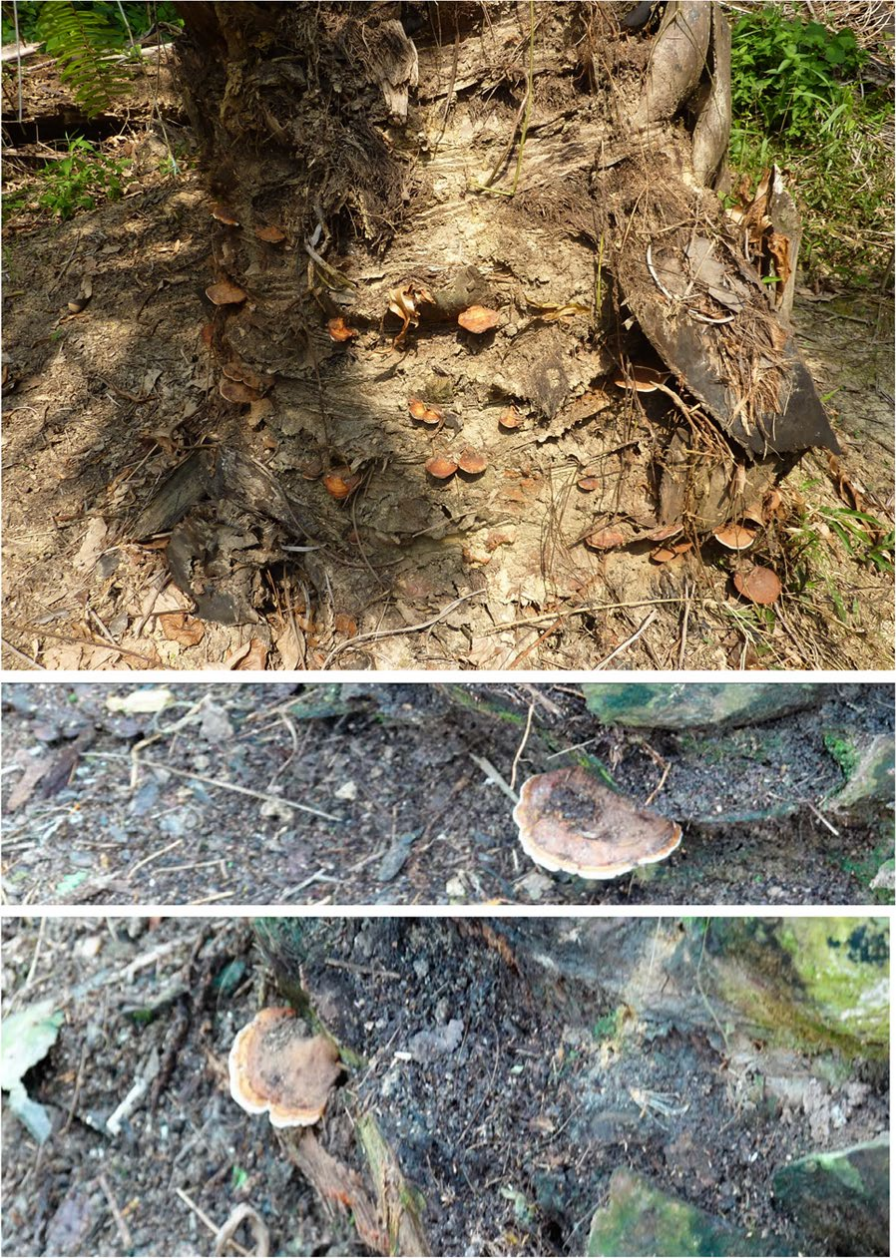
*Ganoderma boninense* basidiomata on oil palm stems. Images are from the authors’ personal collections (RRMP & DM)

*Basal Stem Rot* (BSR) is caused by the fungus, *Ganoderma boninense* (Fig. 1), is a serious disease of oil palm, which can reduce yields by 50–80%. It has increased over recent decades due to its spread from infection foci at a greater rate, following repeated cycles of crop planting on infested sites. In Malaysia, BSR is often reported in young plants and seedlings, whereas previously only mature oil palms were infected (Paterson 2019a, 2020c). By the time an oil palm stand is halfway through its ca. 25-year economic lifespan, BSR can kill 80% of plants. Furthermore, expansion of industrial oil palm cultivation began early in Sumatra, where *G. boninense* adaptation to the environment is most likely to occur. This region contains the highest levels of disease, implying an association between the duration of oil palm cultivation and higher disease concentrations. BSR is found increasingly in inland peninsular Malaysia and Sabah, and in some cases is at high levels in places where it has not previously been detected. BSR was also reported at high levels in oil palm grown on inland lateritic soils and peat soils, irrespective of cropping history, whereas before such soils had been disease-free. By the time of replanting (every 25 years), 40–50% of palms were lost in some fields, with the majority of standing palms showing disease symptoms. This information indicates a trend for increasing BSR with projected climate change. However, the climate for growing oil palm is currently optimal and has been so for many decades. The increase in disease previously reported was from increased virulence of the fungus, rather than increased susceptibility of oil palm due to a less suitable climate.

BSR may increase further by natural selection of more virulent strains and oil palm cannot always adapt rapidly enough to respond to changes in pathogen virulence. The BSR pathogen has the ability to infect oil palm plants at a rate of as much as 80% incidence over half of its economic life span (Corley and Tinker 2015). *Ganoderma* is a variable genus with poorly defined species concepts and will adapt to climate change more readily than oil palm via natural selection of more virulent strains (Mercière et al. 2017). In Indonesia, BSR is less severe in Kalimantan than in Sumatra, probably due to younger crop rotations (Suryantini and Wilandari 2018; Paterson 2019a, b, 2020d). In Thailand, national BSR incidence is relatively low with a reported rate of 1.53%, although it is more widespread in the south (Basal stem rot of oil palm 2020). In Southern Thailand, BSR incidence may be influenced by proximity to peninsular Malaysia where disease rates are also high (Pornsuriya et al. 2013). In Papua New Guinea, BSR incidence is not as high as in other areas of SE Asia, although rates of 50% have been recorded in some regions. An average of 25% infection is a plausible scenario for this country as the initial incidence is lower than in Malaysia and Indonesia. BSR incidence is probably low in Myanmar as the plantations are more recent and distances between them are large. Myanmar has a distinctly different climate to the rest of SE Asia and is less capable of growing oil palm per se (Paterson 2020b).

Paterson (2020c, d) considered BSR in Malaysia and Indonesia respectively and in the regions of the countries. Disease incidence was much higher in peninsular Malaysia than in Sarawak, and especially Sabah. Sabah may therefore be a more sustainable region from the perspective of BSR incidence. In Indonesia, Sumatra and Java had particularly high incidences compared to other areas such as Sulawesi and Papua. These scenarios indicated which regions may be suitable in terms of future sustainability of the industry. However, the environmental effects, especially from deforestation, should be of prime importance in planning new plantations.

*Fusarium oxysporum* f.sp. *elaeidis* (Foe) results in acute and chronic wilt of oil palm particularly in Africa (Paterson 2021e). A major outbreak devastated OP in West and Central Africa where it has a particularly high incidence (Rusli 2017). Foe in Malaysia and Indonesia is controlled by quarantine procedures, although native strains can infect oil palm in vitro. Avoiding introduction from endemic areas is essential to prevent Foe in regions where it is does not normally exist. However, importation of breeding materials from Africa is required to expand genetic diversity in Malaysia and Indonesia, implying a risk from infested seed and pollen. Quarantine procedures in Malaysia and Indonesia are undertaken, although the risk of spread remains, especially because climate change may increase disease (Rusli et al. 2015).

In the Ivory Coast, 20% of palms planted from 1964 to 1967 displayed vascular wilt symptoms, with some crosses at 70% (Cochard et al. 2005). But from 1976 to 1983 vascular wilt rates of < 2% were observed and in the 1990s, it was difficult to find symptoms in plantations. These reductions were attributed to breeding for resistance. Rusli et al. (2015) found that Foe infection of oil palm was frequent in Ghana with incidences of 10.4% and 8.3% and also detected the presence of *Foe* in ca. 11% of symptomless palms in plantations. Decades of selection and breeding for wilt resistance occurred in Ivory Coast where 20% of palms planted from 1964 to 1967 displayed vascular wilt symptoms, with some crosses at 70% (Cochard et al. 2005). Rusli (2017) demonstrated that Malaysian oil palms were susceptible to infection by Foe strains from Africa.

*Phytophthora palmivora* is a fungus-like oomycete and a notorious pathogen of oil palm, causing severe damage in Latin American countries, such as Colombia (Corley and Tinker 2015*).* The disease has recently devastated > 30,000 ha in South West Colombia and > 10,000 ha in the Central Zone, and the rapid increase in the disease may be related to climate change. Acute and chronic forms are found, and it is possible that several different diseases have been described under one name. The acute forms are present in Colombia and Ecuador, with the chronic forms found in Brazil (Corley and Tinker 2015). *P. palmivora* disease of oil palm is unreported in Malaysia and/or Indonesia, although a similar spear rot of oil palm has been reported in Africa and Thailand, which may involve *P. palmivora*. Many other hosts for the oomycete exist in Malaysia and Indonesia (e.g. durian) and, in view of a recent extreme outbreak in Colombia, *P. palmivora* presents a potentially severe threat to Malaysian and Indonesian plantations (Paterson 2020a). More assessments of infectivity are essential given that outbreaks of *P. palmivora* could cause severe problems for major SE Asian oil palm industries.

*Other fungi* Several lesser fungal diseases also cause problems for oil palm (Corley and Tinker 2015). Bunch failure is used to describe oil palm fruit bunches that fail to develop from anthesis to harvest, and the disease can be caused by the basidiomycete *Marasmius palmivorus.* Another basidiomycete, *G. philippii*, is closely related to *G. boninense* but is in fact a trunk rot of *Acacia* trees that is also listed as an oil palm pathogen (Corley and Tinker 2015). This species may become more frequently isolated from oil palm due to climate change. *Phellinus noxius* is a basidiomycete, partially responsible for upper stem rot of oil palm, occurring together with *G. boninense* in some cases. *Haematonectria haematococca* has been implicated in spear rot of oil palm in vitro. Dry basal rot of oil palm is caused by the ascomycete *Ceratocystis paradoxa* (anamorph = *Thielaviopsis paradoxa*), which also has been implicated in oil palm fatal yellowing in, for example, Colombia. *Cercospora ealidis* is widespread throughout Africa and causes *Cercospora* leaf spot. It is infrequent in Asia and is primarily a disease of nursery seedlings and frequently carried forward to plantations where it can survive for a long time (Corley and Tinker 2015). *Glomerella cingulata* is responsible for anthracnose disease in oil palm, although it is not severe currently. All these are diseases of oil palm and it is important to assess how they will be affected by climate change in future studies.

### Pests

In general, pest species of oil palm do not have as much impact on the crop as diseases, with the possible exception of the rhinoceros beetle, *Oryctes rhinoceros*, which emerged as the major pest of oil palm in SE Asia in the 1980s. Although chemical insecticides can be effective, they are expensive, they can affect beneficial insects, and the target organisms may develop resistance. This has led to development of biocontrol strategies, the most effective of which are the deployment of two pathogens of the beetle, namely the entomophagous fungus *Metarhizium anisopliae* and the *Oryctes* virus (Ramle et al. 2005). Both pathogens are specific to rhinoceros beetles and as such will not affect other insects. The *Oryctes* virus appears to be endemic in the beetle population, and deliberate augmentation can increase infection levels to > 75%. *Metarhizium* fungal spores can be applied to areas of infestation as a spray that is highly effective at controlling, but not totally eradicating, the beetles. The combined use of these and other natural pathogens of the rhinoceros beetle have the potential to reduce its harmful impact on the crop, while also minimizing risks of resistance development.

With the projected increase in oil palm replanting over the coming years, it will be important to consider the wider release of such biocontrol agents into areas where rhinoceros beetle incidence is particularly high. These and other forms of integrated pest management are being investigated as primary options in plantations across SE Asia (Ramle et al. 2005; Kalidas 2013). The rapid expansion of high intensity commercial plantations in new regions such as West Africa and South/Central America, plus climatic changes, are likely to result in the emergence of new pests and pathogens. Therefore, it will be important for the public sector and industry to work together in developing improved methods of surveillance and early detection of such threats (Kalidas 2013; Caudwell and Orrell 1997).

## Impacts of climate change

The negative impacts and significance of climate change are well documented in the scientific literature and are now broadly accepted by most of the general public. Climate change threatens the sustainability of crop production via factors such as temperature, rainfall and disease patterns (Rosenzweig et al. 2008). However, the likely effects on tropical crops remain less well known, especially in SE Asia, Africa and Latin America (Ghini et al. 2011; Feeley et al. 2017; Sarkar et al. 2020), although recent research has started to address the situation for oil palm (Paterson 2019a, b, 2020b, c, 2021a, b; Paterson and Lima 2018; Paterson et al. 2015, 2017; Sarkar et al. 2020; Shabani et al. 2012), as discussed below. Climate change effects on natural systems require prediction to mitigate consequential changes in diversity and ecosystem function (Feeley et al. 2017). Mapping of plant disease distributions can influence biosecurity planning, specifying areas that qualify for eradication or containment. The CLIMEX model has been developed for current and future species distribution where knowledge about climate change effects on species distributions is essential in mitigating negative impacts (Lenoir and Svenning 2015).

### Effects of oil palm cultivation on climate change

Koh and Wilcove suggested that oil palm expansion occurs at the expense of forests acting as carbon sinks (Koh and Wilcove 2008). Dislich et al. (2017) determined 11 of 14 ecosystem functions decreased in levels of function by the introduction of oil palm plantations. Fitzherbert et al. (2008) determined that oil palm plantations support many fewer species than forests and some other tree crops: Habitat fragmentation and increased pollution can further increase GHG emissions. The detrimental aspects of increasing numbers of oil palm plantations has been discussed in terms of deforestation and haze production from burning peat soil to clear ground for new plantations (Tonks et al. 2017; Cook 2018; Veloo et al. 2015). These processes release GHGs contributing to climate change (Dislich et al. 2017).

The conversion of tropical rainforests into oil palm plantations is the primary environmental impact of the industry (Paterson and Lima 2018). Forested areas are used for the expansion of plantations where the emissions from conversion exceeded the potential carbon fixing of oil palm (Paterson et al. 2015, 2017). Oil palm production involving deforestation re-leases global anthropogenic emissions of 6–17% CO_2_ (Wich et al. 2012). The highest carbon emitter countries from forest cover loss are Brazil, Indonesia and Malaysia with values of 340, 105, and 41 [Teragrams (Tg) C/year] respectively. Indonesia and Malaysia account for high C emissions from deforestation as they are the first and second highest producers of oil palm. Substantial palm oil production is also undertaken in Columbia and Nigeria (Paterson et al. 2017). Emissions from oil palm cultivation in Indonesia accounted for ca. 2–9% of all tropical land use emissions from 2000 to 2010 (Carlson et al. 2018) and deforestation accounted for about 30% of global warming-related pollution emissions in 2009, with Indonesia as the world’s seventh-largest producer of such emissions. Plantation expansion in Kalimantan, Indonesia contributed 18–22% of the country’s CO_2_ emissions in 2020.

Large reductions in emitted GHGs and climate regulation function occur due to conversion of forest to oil palm plantations (Dislich et al. 2017). More GHGs and volatile organic compounds (VOCs), which are precursors to tropospheric ozone, are produced by oil palm plantations. GHGs emitted from land-clearing fires and land and plantation establishment are significantly greater than carbon sequestered by oil palm. VOCs, GHGs and aerosol particle emissions during fire periods result in direct and indirect changes of solar irradiation while undisturbed forests give lower air and soil temperature and higher air humidity microclimates compared to plantations (Dislich et al. 2017).

Indonesia has increased oil palm plantations, reducing drastically the primary forest. Sumatra has the highest primary rainforest cover loss in the country. Forest cover in Riau and Jambi declined from 93 to 38% between 1977 and 2009 changing microclimatic conditions from lack of natural forests regulation. Warming of land surface and increases in air temperature from climate change occur from oil palm expansion as observed in Sumatra (Paterson and Lima 2018). Oil palm foliage cover is lower, more open, and simpler than tropical rainforest foliage cover. Warming occurs from reduced evaporative cooling and warming induced by land cover change (LCC) exceeded the global warming effect.

The predominant compound contributing to the GHG from oil palm plantations is CO_2_ whereas nitrous oxide and methane are at reduced concentrations, although with greater effect per molecule. Large releases of CO_2_ from land-clearing fires occur, particularly on peat. Also, fires indirectly increase emissions by increasing peat decomposition. Drainage of peat soil releases large concentrations of CO_2_ to establish plantations by oxidation and decomposition: dissolved organic matter is removed from peat soils during drainage, which decomposes and releases additional CO_2_.

The very high fruit production of oil palm allows greater assimilation of CO_2_ and produces more biomass than forests and is often used erroneously as an argument in favour of oil palm. This higher rate of C uptake does not compensate for that released when forests are cleared, as forests have more biomass than oil palm plantations unless very long timescales of hundreds of years are considered, well beyond the maximum time frame of ca. 80 years considered in Paterson et al. (2015, 2017) in terms of the likely effect of climate change on suitable climate for oil palm growth for example.

Fires also add black carbon (soot), which increase global warming and oil palm plantations release more N_2_O into the atmosphere than forests, mainly from fertilizer use. Peatland deforestation for oil palm cultivation in West Kalimantan, Indonesia, increased GHG emissions greatly (Paterson and Lima 2018). Overall, the biological and managerial tools to surmount many challenges exist but need much better support (Murphy 2014) and will be discussed below. In addition, large scale conversion of tropical forest to oil palm plantations has detrimental effects on biodiversity.

### Effects of climate change on oil palm cultivation

In terms of general effects, climate change is likely to affect sustainable production of palm oil as climatic suitability will decrease, with concomitant increases in economic and social problems in producing regions. Poleward movements in climate-related ranges of particular plants are by far the most frequently reported, including limited reports on poleward change in suitable climates for oil palm growth (Paterson et al. 2017; Fei et al. 2017). How species may react under climate change has been reported including the detrimental effect on the suitability of future climates on oil palm growth in a global setting (Paterson et al. 2017). Furthermore, oil palm production creates climate change as discussed above and this will affect detrimentally the ability to grow oil palms and alter their distribution (Paterson and Lima 2018). Oil palm is currently grown in optimal climatic conditions and has been for many decades (Corley and Tinker 2015).

**Fig. 2:**
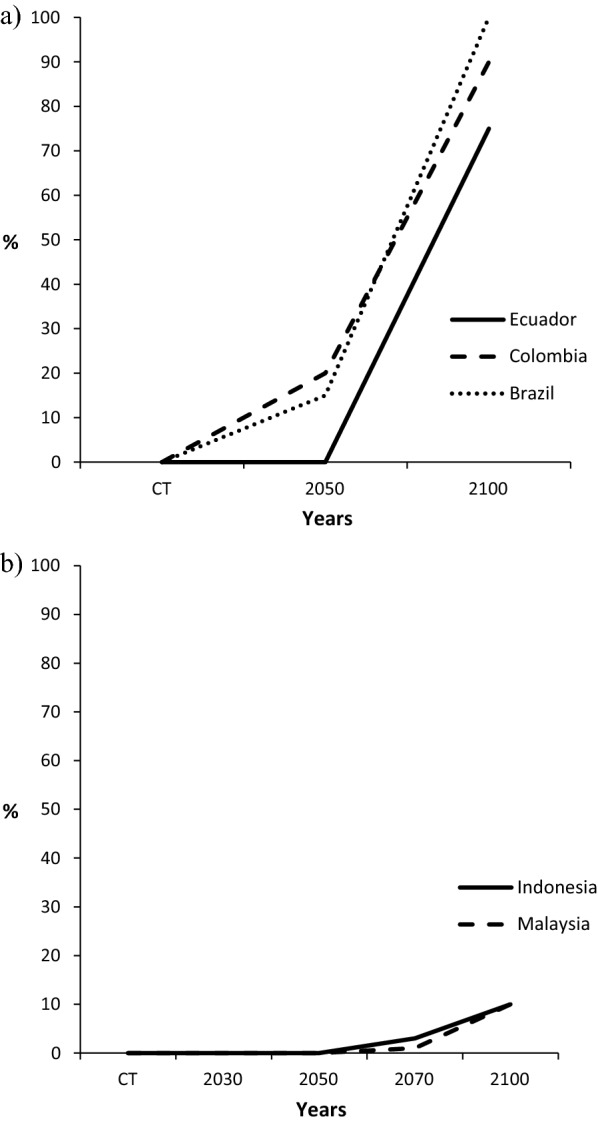
**a** Oil palm mortality in Ecuador, Colombia and Brazil. **b** Oil palm mortality in Malaysia and Indonesia. These data take into account projected future changes in suitable climate for growing oil palm. (Adapted from data provided in Paterson (2021b)

Suitable oil palm climatic impact data have been used to create schemes for its mortality by postulating that large degrees of unsuitable and marginal climates in particular were likely to cause high amounts of mortality. Also, reductions in highly suitable and/or suitable climate per se would not cause a significant effect on oil palm mortality. Simulation modelling to determine suitable climate scenarios for growing oil palm (Paterson et al. 2017; Paterson 2019a), were employed to estimate how climate suitability for oil palm growth would change the estimated mortality rate from unsuitable climatic conditions. Predicted percentage oil palm mortality was determined in (a) SE Asia (Paterson 2020b) and (b) Latin America and extrapolated to Malaysia and Indonesia (Paterson 2020c) (Fig. 2a, b). These percentages represent large numbers of oil palms in Malaysia, Indonesia, Thailand and Papua New Guinea because of the large numbers grown in these countries. Information on oil palm mortality is also provided for some African countries in Paterson (2021e).

The detrimental effect of future climate changes on oil palm cultivation globally and on oil palm mortality in Kalimantan, Indonesia and some other SE Asian countries were determined (Paterson 2020b, c; Paterson et al. 2017), which provided information relevant to Malaysia (see Table 3). High oil palm mortalities were predicted for Thailand, and Myanmar and low mortalities for Kalimantan and the Philippines, while Papua New Guinea was intermediate (Harris et al. 2013; Gunarso et al. 2013). Modelling of oil palm mortality for three South American countries, Malaysia and Indonesia was performed (Paterson 2020c) using similar methods to Paterson (2020b). The Latin American countries, particularly Brazil, were assessed to have high future mortalities, whereas the figures for Malaysia and Indonesia were much lower. These potential effects on mortalities will have detrimental consequences on future abilities to meet the demand for palm oil. High levels of mortality were determined for Peninsular Malaysia but not in Sabah or Sarawak in the future from unsuitable climate (Paterson 2020c). In Indonesia, regions such as Sulawesi and Papua had low levels of mortality in contrast to Sumatra and Java where high mortality was determined (Paterson 2020d). A study of predicted oil palm mortalities in South America found that by 2050, low mortalities are predicted in (a) the East Coast from Brazil to Suriname, (b) more centrally in Paraguay and (c) Colombia, Peru and Ecuador in the west. High mortalities were determined for Guyana, Bolivia, Western Brazil and Venezuela (Paterson 2021a). By 2100, much higher mortalities were determined for all countries except Paraguay, which appeared virtually immune to the effects of future climate. Very high mortality of oil palm was determined for Ghana and Nigeria in Africa, especially by 2100 (Paterson 2021e), whereas Cameroon had low levels,

**Table 3:** Predicted oil palm mortalities (%) with climate change in various South American and SE Asian countries, plus the Kalimantan province of Indonesia. Adapted from data provided in Paterson (2020b)

Countries	Years		
	2050	2070	2100
Indonesia	0	3	10
Malaysia	0	1	10
Kalimantan	0	5	10
The Philippines	0	ND	10
Papua New Guinea	0	ND	40
Myanmar	5	ND	60
Thailand	10	ND	70
Colombia	20	ND	90
Ecuador	0	ND	75
Brazil	15	ND	100

*ND* not determined

African oil palms are likely more badly affected by climate change from increased GHGs, although there appears a low extinction risk in the immediate future. Furthermore, losses of oil palm habitats such as tropical rain forests are exacerbating the pressures on oil palm populations: their ecosystem functions and services will be highly sensitive to climate change. Blach-Overgaard et al. (2015) predicted climate suitability losses across almost all regions where palms occur in Africa and CLIMEX modelling indicated that Africa will have less suitable climatic conditions for oil palm cultivation (Paterson et al. 2017). However, sharp longitudinal trends to potential refuges from west to east Africa were found, which could allow oil palm to survive naturally, or by the creation of new plantations towards to east of the continent, with, of course, environmental concerns being paramount (Paterson 2021a). Using similar methods, a phased increase in suitable climate was predicted, which implied more unsuitable climate for growing oil palm towards the centre of the South American continent (Paterson 2021b). Increasing longitudinal trends in suitable climate for growing oil palm SE Asia were observed from current time to 2050 and 2100 from west to east (Paterson 2021c). Paterson (2021d) developed an improved model for determining suitable climate for growing oil palm in Africa which confirmed the west to east trend and could be employed in other regions such as South America and SE Asia. 

A significant negative relationship was found between annual average temperature and sea level rise and oil palm production in Malaysia temperature with rises of 1 to 4 °C potentially causing oil palm production to decrease by 10 to 41% (Sarkar et al. 2020). Future changes to suitable climates for growing oil palm worldwide were considered using modelling based on temperature, soil moisture and wet stress data (Paterson et al. 2017). The general predictions were for a reduced level of suitable climatic regions by 2050 and further reductions by 2100. The projections indicate serious consequences to the oil palm industry generally. In Africa, the climate is predicted to be less suitable for growing oil palm at the same rate, or faster than, Malaysia and Indonesia with the exception of Uganda where increases in climatic suitability were predicted. Paraguay appears to gain suitability in climate for growing oil palm in South America, whereas Venezuela will have a particularly low level of suitable climate. French Guiana, Surname and Guyana appear to maintain suitable climates and large losses were determined in west Brazil by 2100. The western countries of Colombia, Peru and Ecuador will suffer severe losses of suitable climate. Furthermore, there was a three-phase trend in suitable climate rather than a single direct longitudinal change (Paterson 2021b). Vietnam, the Philippines, Papua New Guinea (PNG) and island Malaysia had increased suitable climate by 2050 in SE Asia. Large decreases in suitable climate by 2050 for Thailand, Laos and Cambodia, which are towards the west of SE Asia, were observed (Paterson 2021c).

Climate has an important role in defining the range limits of oil palm distribution by exerting eco-physiological constraints (Paterson et al. 2017). However, factors such as soil properties and biotic interactions may prevent plants from colonizing sites that are otherwise suitable. Studies such as Paterson et al. (Razak et al. 2020) are unusual in that a wider range of climatic conditions are considered than only temperature (Paterson 2021a). Changes in climate will have broad-scale impacts on the distribution of oil palm. Alterations in cold, heat and dry stresses were largely responsible for the changes in climatic suitability for oil palm cultivation, while wet stress was unimportant, hence extending the range of parameters from temperature alone (Paterson et al. 2017). Apart from temperature (Feeley et al. 2017) and diseases, a wide range of factors still awaits consideration, although studies on effects on crop production have been reported (Lobell et al. 2006).

One of the most important future threats is the emergence of new pests and diseases and/or the movement of existing diseases from one part of the world to another. The transfer of existing biotic threats could occur due to climatic factors, but another mechanism is movement via trade, travel or other human agency where potential pathogens might elude current biosecurity measures. For example, the serious impact of *P. palmivora* on plantations in S. America, and if this pathogen were to reach the central growing regions of SE Asia, its impact could be devastating (Paterson 2020a; Mohamed Azni et al. 2019). Although biosecurity measures are already in place, these tend to be focussed on known threats and may not be able to cover all of the many potential entry routes for a new pathogen. A similar situation exists for Fusarium wilt disease of oil palm (Paterson 2021e) with African countries suffering most from this particular disease.

An example of an unexpected new form of pathogen of oil palm is the orange-spotting coconut cadang-cadang viroid variant (OSCCC-Vd) (Coconut cadang-cadang viroid (cadang cadang disease) 2020). Viroids were only discovered in the 1970s and are the smallest and simplest known type of infectious pathogen, consisting of just one small, naked, circular single-strand of RNA that does not encode any proteins. The origin of viroids is unknown with some suggestions that they might date from an ancient non-cellular ‘RNA world’, although a more parsimonious hypothesis is that they have arisen de novo on multiple occasions as plant-specific pathogens (Catalán et al. 2019). OSCCC-Vd normally infects coconut plantations and is endemic in the Philippines, but early in 2011 a putative variant was found in oil palm plantations in Sabah, triggering a ban on the movement of oil palm materials to other parts of Malaysia. Although the threat of OSCCC-Vd eventually receded in the 2010s, this episode exposed problems in the surveillance mechanisms and phytosanitary procedures in the face of the sudden appearance of a hitherto unknown pathogen.

The effects of climate change on oil palm diseases by fungi and by the oomycete *P. palmivora* have been discussed and indicate a trend for increased BSR, Fusarium wilt  and *P. palmivora* incidence with climate change. Modelling of the effect of changes in climate on the infection levels of BSR in Sumatra, Indonesia, including quantitative BSR data, indicated that BSR would become even more serious after 2050 (Paterson 2019b). Weather is a major factor in crop pathogenesis and, when crops suffer cold, heat or desiccation stress, they may be more susceptible. Mountain areas were considered in this assessment which affected some results considerably. For example, hilly regions in North Sumatra did not provide a suitable climate for oil palm.

**Fig. 3:**
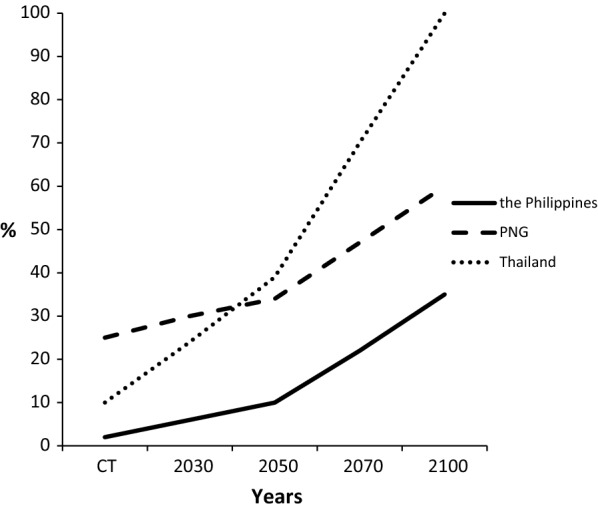
Basal stem rot in three S E Asian countries. The incidence of disease was determined from the changes in suitable climate for growing oil palm as described in Paterson (2020b)

A similar ‘Agriculture 4.0’ methodology of big data and simulation modelling was used to produce a scheme of how BSR might advance under future climates in Malaysia (Paterson 2019b). The assessments of BSR were merely qualitative and indicated, nevertheless, that the levels of infection would also increase a great deal after 2050. Paterson (2020b) considered future climate effects on BSR in Kalimantan and alternative countries in SE Asia. Kalimantan and the Philippines were assessed as sustainable, but Thailand and Myanmar were unsustainable, while Papua New Guinea was intermediate in sustainability (Fig. 3). *P. palmivora* is prevalent in South America and Paterson (2020c) extended the principles described above to the disease. Colombia and Ecuador were highly susceptible, while Brazil was less so. However, a severe threat to Malaysia and Indonesia was assessed, which would require increased future vigilance to control the disease. Paterson (2021e) indicated an equivalent situation for Fusarium wilt of oil palm focusing on African countries extrapolated to Malaysia and Indonesia.

### Amelioration of climate change effects on oil palm and vice versa

Procedures for amelioration of the effects of climate change on oil palm and the effect of oil palm on climate change, have been discussed as partially based on CLIMEX models (Paterson and Lima 2018). The situation with the oil palm industry cannot be business as usual in light of the effects of climate change on oil palm and vice versa. A series of procedures have been devised to address how the industry might mitigate these problems (Paterson and Lima 2018). Many of these measures will help to maintain the biodiversity normally associated with forests because they will stop the plantation being a monoculture. Also, the soil microfauna will likely increase as a result of these measures.

#### Reducing the effects of oil palm on climate change

Plantation management measures can prevent or reduce losses of some ecosystem functions which will reduce climate change. These include (a) avoiding illegal land clearing by fire, (b) avoiding draining of peat, and (c) using cover crops, mulch, and compost (Dislich et al. 2017). Reducing GHG by limiting oil palm expansion to areas with moderate or low carbon stocks is most effective. This involves ceasing development of plantations on peatland and enforcing the moratorium on new concessions in primary forests. In addition, rehabilitation and restoration of converted peatlands are an option. Limiting the problems of flooding may prevent increased CH_4_ emissions on mineral soils. Reducing unnecessary expansion of plantations and ensuring existing ones are managed optimally are crucial. Mechanisms such as (a) reduced emissions from deforestation and forest degradation, plus conservation, sustainable management of forests, and enhancement of forest carbon stocks (REDD+), (b) national greenhouse gas accounting, and (c) accurate emission factors for C dynamics are essential (Comeau et al. 2016). Considerable funding has been obtained for REDD + scheme. REDD + proposals include growing oil palm on reclaimed soil and replacing the use of fertilizer with other methods. A few plantations are replacing grassland or scrub where the average C content of the plantation will exceed that of the previous vegetation and so becoming a greater C sink.

Controlling disease may assist in decreasing the unwanted expansion of plantations as yields will be increased from reduced disease in current plantations, such as described for *Ganoderma* rots of oil palm (see below). The current awareness of environmental issues makes optimizing current plantations by reducing disease imperative in any case. Reducing nitrogen fertilizer is an effective way to decrease nitrogen-based emissions (Dislich et al. 2017). Oil palm plantations release large quantities of nitrous oxide (N_2_O) into the atmosphere linked to nitrogen (N) fertilizer use. An option for oil palm planting, without threatening tropical rain forests, is the rehabilitation of anthropogenic grassland that was created by human clearance of natural forest many centuries ago. For example, there are extensive areas of anthropogenic grassland in Indonesia where much of the spread of oil palm plantations will take place (Paterson and Lima 2018).

#### Reducing effects of climate change on oil palm

Evidence is growing of the existing and likely future impacts of anthropogenic climatic changes on the oil palm industry. Immediate priorities should include further research to understand climatic effects on oil palm in the many regions of the tropics where the crop is now grown, and to begin the implementation of mitigation strategies to minimize adverse effects. Most climatic threats identified to date involve periods of elevated temperature and reduced rainfall, both of which cause stresses that impact on overall crop performance, and in particular oil yield. Increasingly well documented impacts of climatic cycles such as El Niño and La Niña have underlined the crucial role of climate for oil palm performance and oil yield (Rahutomo 2016; USDA 2016).

Strategies are required to minimize the adverse effects of climate change on oil palm cultivation: it cannot be business as usual for the industry. These practices may also decrease climate change from reduced deforestation if the yields of existing oil palm are optimized to cope with climate change. More dispersed cultivation outside the main producing countries could ameliorate threats from climate change as a wider range of climates would be encountered, some of which may be more suitable for oil palm. Current expansion into West Africa and South/Central America already underway was intended to create a more secure production system in the longer term. However, Latin America and Africa may be even more affected by climate change in terms of suitable climate for growing oil palm than SE Asia, implying that expansion will be unlikely. Even within these continents there are trends which will be useful for plantation managers (Paterson 2021a, b).

Cultivation at higher altitudes and/or lower and higher latitudes may be possible beyond the lowland tropics as climate change progresses. An increase in highly suitable climate for growing oil palm by 2030 in Indonesia and Malaysia largely in mountainous regions of Sumatra, Sarawak, Borneo, and Sulawesi was reported (Paterson et al. 2015). There may be novel areas for oil palm development even under climate change, although in general, the climate suitability per se will be reduced. A caveat being potential biodiversity and ecological function loss if novel areas are converted from, for example, forest. The use of cover crops to reduce climate effects on oil palm may be possible and increases biodiversity. The sustainability of oil palm production will depend in part on using cover crops, especially under suboptimal conditions. Leguminous cover crops are grown to (a) coexist with oil palm following jungle clearing and planting/replanting, (b) provide complete cover to an otherwise bare soil, and (c) protect from erosion. They also perform multiple functions such as reducing soil water evaporation, reducing runoff losses, improving and maintaining soil fertility, and recycling of nutrients (Samedani et al. 2015). They promise reduced environmental pollution and improved crop yields. Legumes may reduce C and N losses from oil palm systems and increase soil C sequestration. Some examples for oil palm are as follows: Pigeon pea (*Cajanus cajan*), Calopo (*Calopogonium mucunoides* Desv.), butterfly pea (*Clitoria ternatea*), white tephrosia (*Tephrosia candida*), and Brazilian stylo (*Stylosanthes guianensis* var. *guianensis*) some of which are already in use in SE Asia (Paterson and Lima 2018). The biodiversity of the plantation will be increased per se as each plant is introduced and by the increase in nitrogen fixing bacteria associated with the legume.

Soil management practices including (a) empty fruit bunch (EFB) application, (b) palm frond application and chemical fertilization improving soil fauna (worms, beetles, and ants) feeding activity, and (c) better soil chemical properties, all of which show considerable promise. EFB applications greatly enhanced soil fauna feeding activity and are associated with increased concentrations of base cations and soil moisture. This elevated biological activity has good potential to assist ecosystem functions such as litter decomposition, nutrient cycling, organic carbon stabilization, and ultimately oil palm productivity. The use and presence of earthworms may increase the effectiveness of growing oil palm, as they can contribute to soil turnover, structure formation and serve as a fertility enhancer and, again, increase biodiversity (Paterson and Lima 2018).

Developing new oil palm varieties resistant to climate change is another possibility (Rival 2017), although may not be easily achieved. Breeding oil palm for climate change requires multidisciplinary and collaborative research at a high level (see next section). Selecting for complete resistance, rather than tolerance to diseases, leads to high selection pressures for new variants of the pest/pathogen that can overcome the resistance in the crop. Resistant oil palm cultivars to climate change, or environmental stress, may overcome the less favourable growth conditions imposed by climate change (Tang 2019). However, it is impossible to know accurately what climate changes will be needed to enable resistant cultivar development, e.g. a cultivar resistant to desiccation stress may be sensitive to high temperature. High fertilizer use causes increased emissions of GHG from manufacturing, transportation, and application, and improvements will be required in oil palm nutrient uptake efficiency by breeding for suitable root systems.

Methods that ameliorate the effect of (a) climate change on oil palm and (b) oil palm cultivation on climate change include the following: Optimizing the rhizosphere by adding arbuscular mycorrhizal fungi (AMF) will also assist in reducing climate change with generalized benefits to oil palm growth, by reducing the need for fertilizer for example. Arbuscular mycorrhizal (AM) symbioses have beneficial effects on water transport to assist in overcoming drought conditions, of relevance particularly to ameliorating climate effects. Reducing fertilizer production and use will cause decreased emissions that lead to climate change, and the use of AM could ameliorate the effects on oil palm. AM and AMF addition will increase biodiversity within plantations (Paterson and Lima 2018).

“Slash-and-char” as an alternative to “slash-and-burn” of forests cleared for oil palm may be beneficial and feasible. Slash-and-char effectively produces charcoal to sequester CO_2_ normally employed for forest residues. This could be used more extensively to improve agriculture in the humid tropics, enhancing local livelihoods and food security, while sequestering various forms of carbon (C) to mitigate climate change. Biochar soil management systems can deliver tradable C emissions reduction as the C sequestered is accountable and verifiable. The fraction of the maximum sustainable technical potential that is realized will depend on socioeconomic factors, including the extent of government incentives and the emphasis placed on energy production relative to climate change mitigation (Paterson and Lima 2018). Reduced tillage is another possibility for affecting climate change, where reducing tillage with AMF provides the optimal conditions for oil palm. Low tillage combination with AMF assists nutrient uptake, water relations, and protecting against pathogens and toxic stress, hence potentially ameliorating the effect of climate change on oil palm growth. Also, low tillage will decrease the emission of GHG from oil palm plantations (Paterson and Lima 2018).

An important tool used by policymakers to assess the impacts of a particular cropping system is life cycle assessment (LCA) (Schmidt 2015; Yee et al. 2009). This method seeks to estimate the impact of all aspects of the production process from planting seed, growing, harvesting and processing the crop (including fuel and labour costs); application of inputs such as water, fertilizer, herbicides, and pesticides; shipping of the oil overseas and downstream conversion into products such as foods and oleochemicals; transport to wholesalers, retailers, and consumers; and finally, disposal of products at the end of their lifetimes. Unfortunately, very few published studies cover the entire system ‘from cradle to grave’.

The most effective manner of addressing climate change is to adhere to policies devised at the 2019 COP25 climate meeting by reducing GHGs and future temperature rises. Conservation scientists, managers and environmental policymakers need to adapt their guidelines and policies to mitigate the impact of climate change (Brooke 2008). The new recommendations from COP meeting in Glasgow, Scotland in 2021 should be implemented as a matter of urgency as the most effective procedures for controlling climate change and consequently the effects of climate change on oil palm. Importantly, palm oil producers should also collaborate more effectively to help shape future policies on climate change and oil palm.

## Breeding and biotechnology to improve oil palm as a crop

Recently, there have been several significant advances in breeding and biotechnology use for oil palm improvement. This is despite the challenges posed by the long-lived perennial nature of oil palms, which are large plants typically grown commercially for > 25 years. Hence, such biological strategies are much more complex and lengthier to implement compared to the smaller, faster growing annual crops. Breeding efforts have tended to focus on major economic traits such as oil yield and composition, pest and disease resistance, and plant architecture. Until relatively recently, oil palm breeding was also disadvantaged by the restricted genetic pool of commercial varieties, most of which were derived from small numbers of plants imported from Africa to SE Asia in the nineteenth and twentieth centuries. The available gene pool has now been greatly expanded, largely thanks to a series of germplasm collection expeditions to Africa and South America by pioneering breeders such as Rajanaidu et al. (2014). This has now allowed for genome-wide association studies (GWAS) of key traits such as oil yield and fatty acid composition (Ithnin et al. 2020) in the case of American oil palm and an *Elaeis oleifera* × *Elaeis guineensis* hybrid (Osorio-Guarín et al. 2019). Recent breeding-related reviews include genomics, genomic selection (Nyouma et al. 2019), transgenics (Costa et al. 2012), genome editing (Yarra et al. 2020), and marker-assisted selection (Ting et al. 2018; Babu et al. 2019). Following the publication of the oil palm genome sequence in 2013 (Singh et al. 2013), several detailed linkage maps have now become available for the use of breeders (Ong et al. 2019; Herrero et al. 2020).

Genomics-based strategies such as marker-assisted selection are already generating several useful advances for a variety of important traits that include oil yield, fatty acid composition and crop morphology (Xia et al. 2019; A quantum leap with genome select 2020). One of the most exciting recent developments was the announcement in mid-2020 of new breeding lines that are capable of more than double the current average oil yield (A quantum leap with genome select 2020). These plants are part of a genomics-based programme called ‘Genome Select’ carried out by plantation company Sime Darby, with a claimed 9.9 t/ha average yield over 5 years in field trials under optimum conditions. Given that current average palm oil yields are less than 4 t/ha, and that soybean and rapeseed only yield 0.3 and 1.2 t/ha respectively, this could be a game changer for the industry if two important conditions are met. Firstly, the experimental lines will need to be assessed for their oil yield performance under commercial plantation conditions in a range of geographic regions and, if necessary, crossed with locally adapted varieties. Secondly, the new higher yielding varieties need to be part of an ambitious replanting programme that will potentially replace a significant proportion of the estimated 2.5 billion oil palms that are currently under cultivation worldwide.

In terms of molecular genetics approaches to BSR mechanism and control, *G. boninense* genome and transcriptome data are now available with two *G. boninense* genome assemblies in the NCBI Depository (Wong et al. 2019), which provides a table listing publicly available genome and transcriptome data associated with the *G. boninense* and *G. boninense*-oil palm pathosystem. High-throughput next-generation sequencing and improved bioinformatics analyses has greatly facilitated *G. boninense* pathogenesis and housekeeping candidate gene identification. However, *G. boninense* remains poorly studied with respect to system-level gene function studies and biotechnology manipulation, with no available gene co-expression network models. Most studies have focused on host transcriptome data, whilst similar studies on the pathogen remain scarce. Ho et al. (2016) utilised mass RNA sequencing and de novo assembly of RNA-seq and were able to detect a high number of *Ganoderma* transcripts involved in lignin metabolism, such as manganese peroxidase and laccases. It is encouraging that, very recently, in silico mapping within an oil palm breeding program has revealed several QTL associated with genetic resistance to *G. boninense* (Daval et al. 2021).

Publication of the transcriptome of *G. boninense* at monokaryon, mating junction and dikaryon stages (Ho et al. 2016; Daval et al. 2021; Govender et al. 2020) will be useful for investigation of the mating process of this fungus. However, annotation and functional studies of these differentially expressed genes at different stages have not yet been done. RNAi as a tool for functional genomics to study developmental or virulent genes is lacking, although the genome has been sequenced, and there is the promise of new approaches to molecular breeding using genome editing technologies such as CRISPR-Cas-9. The role of genes involved in ergosterol biosynthetic pathway in *G. boninense* utilizing RNAi-mediated gene silencing is currently being investigated (Govender et al. 2020). The identification and verification of candidate genes are crucial for the application of these targets in RNAi-based crop protection, such as host-induced gene silencing (HIGS) or spray-induced gene silencing (SIGS). In addition, a study on the potential application of RNA silencing targeting DCL genes of *G. boninense* to confer protection against basa 581 stem rot is in progress (Govender et al. 2020). The availability of *G. boninense* genome data in public database (NCBI) enables potential candidate genes to be identified for testing and designing of efficient silencing constructs to avoid off-target transcripts, whilst the availability of the oil palm genome data helps to ensure the silencing constructs do not target and negatively affect the host. Because *G. boninense* on attacks oil palm by degrading lignin (Fig. 1), there is the possibility of modifying lignin to make oil palm plants more resistant. Alternatively, making the plant more resistant to initial fungal colonization by inhibiting carbohydrate metabolism first is a more logical approach that possibly overrides the emphasis on lignin per se (Govender et al. 2020).

## Global supply chains and consumer sentiment

Palm oils are globally traded commodities with lengthy and complex supply chains, which can impede implementation of sustainability criteria, such as no-deforestation (Lyons-White and Knight 2018). This complexity is further increased by non-economic factors including sustainability, traceability, disease monitoring and pest management. More recently, however, a more constructive dialogue has emerged as several NGOs and community groups have joined with bodies such as RSPO and some major industry players in exploring initiatives such as certification schemes, that seek to guarantee that palm oils are sourced from sustainable and environmentally friendly sources (RSPO 2009).

### Palm oil supply chain and traceability

**Fig. 4:**
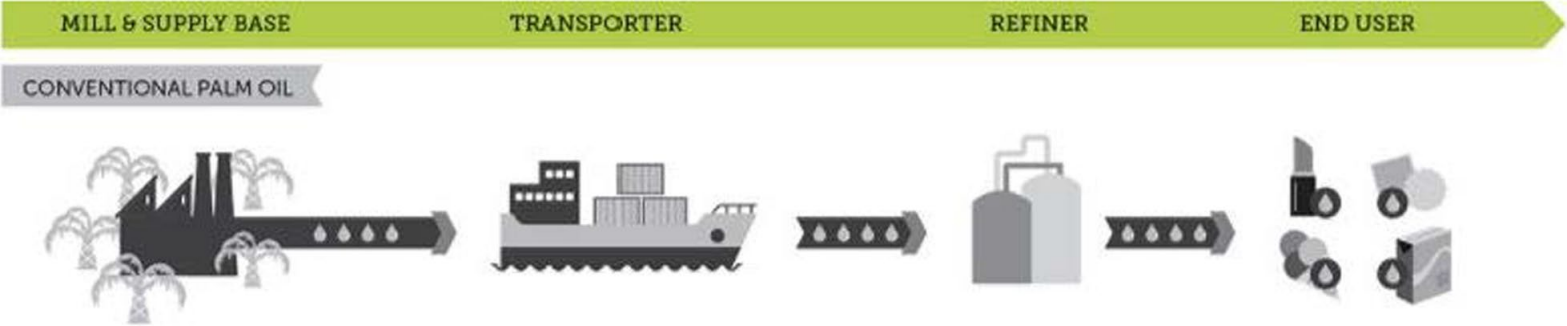
A conventional palm oil supply chain with no certified traceability. The palm oil is produced, transported, refined, incorporated into products and then used by the customer

**Fig. 5:**
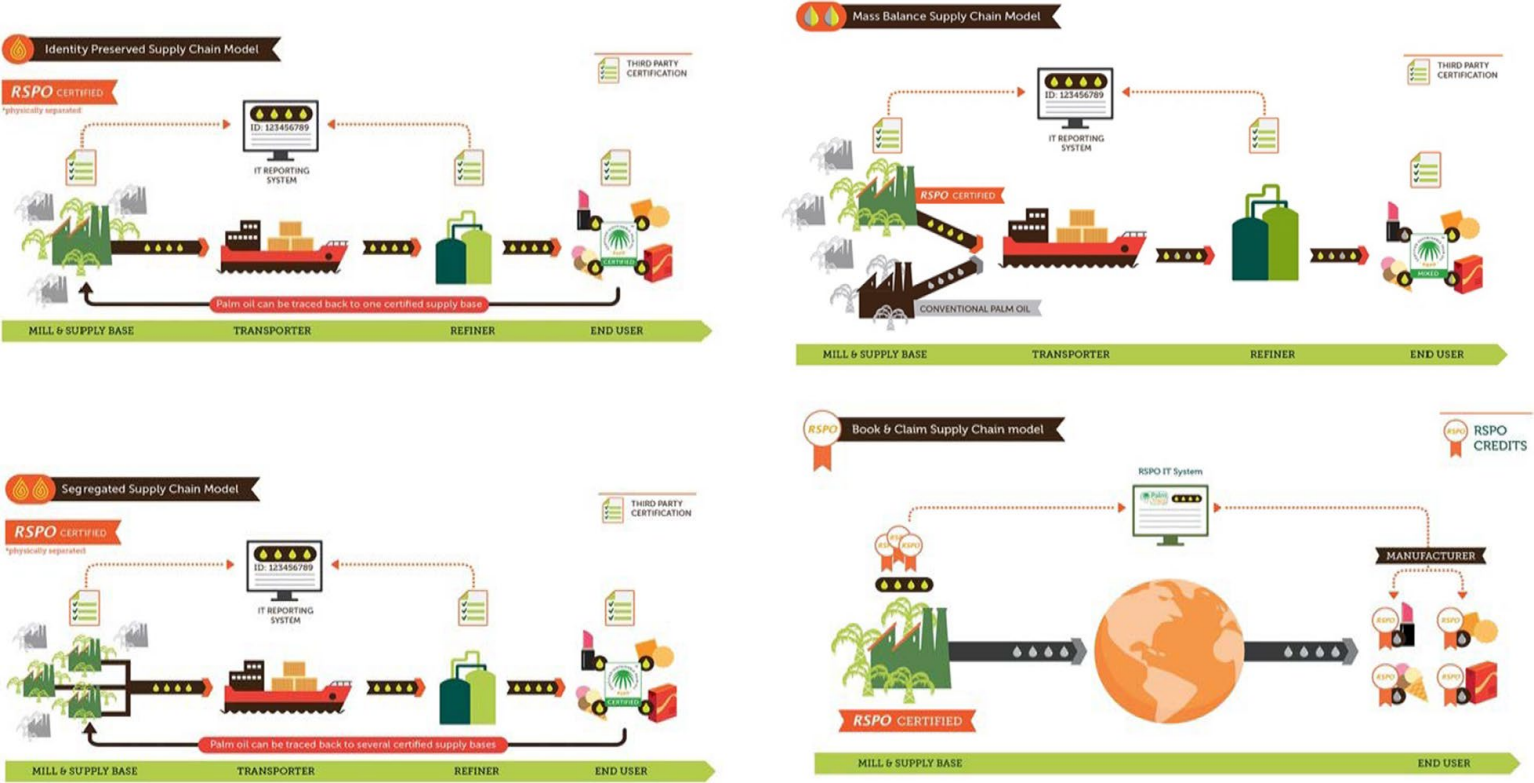
The four different RSPO supply chain models including Identity Preserved, Mass Balance, Segregated and Book and Claim (source: www.rspo.org). The premise of how each supply chain works is described in-text. All palm oils produced under RSPO certification are able to carry RSPO branding, though in the case of Mass Balance and Book and Claim, there is no guarantee that 100% sustainable palms oils are being used

Due to increasing awareness of the wider impacts of oil palm crops, sourcing of palm oil from verified, certified sustainable/responsible sources is of growing interest. Supply chain traceability ensures that information about products can flow easily and enable consumers to have maximum information about product origins. Certification schemes have mostly been established to improve sustainability within the industry, but for these to operate openly and transparently, supply chain traceability is an essential requirement. An overview of a typical palm oil supply chain is displayed in Fig. 4. The most widely used sustainable certification scheme, which aims to improve traceability, is RSPO (RSPO Supply Chains 2017). A graphical overview of each model is also displayed in Fig. 5:

1. Identity Preserved (IP): sustainable palm oil is derived from a single source and kept separate from all other sources throughout the entire supply chain
2. Segregated (SG): sustainable palm oil is derived from multiple sources and mixed; it is then kept separate from conventional palm oil throughout the supply chain
3. Mass Balance (MB): sustainable palm oil is mixed with palm oil from non-certified sources in a controlled and regulated manner
4. RSPO credits: the supply chain is not monitored for the presence of sustainable palm oil. But manufacturers and retailers can buy credits from RSPO-certified growers, crushers and independent smallholders

### Labelling, health and nutrition

Labelling has been shown to influence consumer purchasing habits and to have positive impacts on food production. RSPO believes that using its certification trademark on products will be central to raising awareness and driving demand. Palm oil is used for cooking and is also added to many ready-to-eat foods. Its taste is considered savoury and earthy, with some people describing its flavour as being similar to carrot or pumpkin. It has been a staple in West African and tropical cuisines for millennia (Corley and Tinker 2015). In recent years, the public debate on the health and sustainability of palm oil and its use by food industries has strongly influenced consumer choices. There has been a perception that palm oil, with its relatively high saturated fat content, has adverse nutritional qualities, despite its long history as an important indigenous foodstuff in the tropics. This perception has been strongly challenged by recent meta-analyses and prospective observational studies, mainly conducted in North America and Europe, that failed to demonstrate a correlation between total saturated fat intake and an elevated risk of cardiovascular disease (Chowdhury et al. 2014).

Production of sustainable palm oil is recommended so that consumers only buy from companies using palm oil certified under RSPO, or similar certification schemes that have transparent commitments to improved ecosystem services and human wellbeing (Ayompe et al. 2021). Certification schemes improve consumer confidence and provide a high level of guarantee that that areas of high conservation value are preserved, local communities are supported, and that palm oil plantation managers are implementing best practices including for sustainability and the fair use of labour (Carlson et al. 2018; Schoneveld et al. 2019; Furumo et al. 2020; Santika et al. 2021). Whilst some groups have criticized certification schemes for not moving far or fast enough, researchers and NGOs such as WWF are working with schemes like RSPO, to facilitate greater progress and to include more progressive criteria for best practice, in order to certified. An example of such developments was the announcement in mid-2021 of a multi-stakeholder initiative called Project Lampung (Bootman 2021). This was launched with the aim of linking smallholder farmers in the Lampung province of Indonesia with the NGO, Solidaridad, plus multinationals (including BASF, Cargill and Estée Lauder) in order to enable their palm oil to reach global markets via RSPO certification (RSPO 2021).

## Future prospects

As with many other sectors of commercial agriculture, the global oil palm industry is currently facing significant future challenges as it comes under increased scrutiny in an increasingly interconnected world. Many of these issues, such as the future of palm-based biodiesel, the stagnation in crop yield and related labour problems, and concerns about sustainability and environmental impact are relatively longstanding, but they have been brought into sharper focus as a consequence of the COVID-19 pandemic that started in 2020 and is likely to have long-term effects on the industry as will now be discussed.

### An uncertain future for palm-based biofuels

**Fig. 6:**
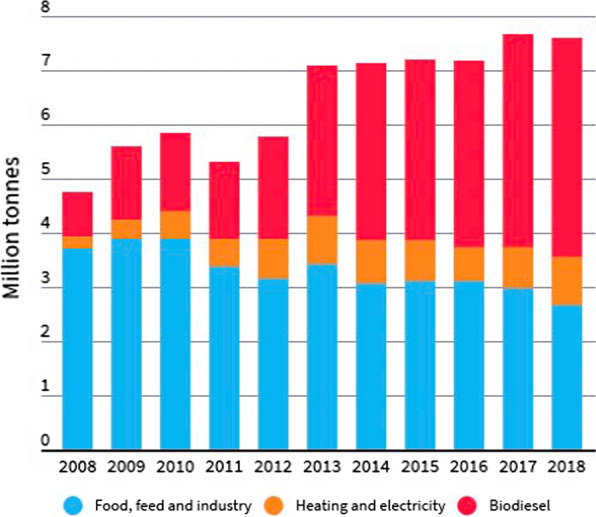
EU palm oil consumption by end use. A steady decline in food use is mirrored by an increase in biodiesel use for palm oil imported into the EU from 2008 to 2018. Source: Ref. Muzii (2019)

Over the past decade a growing proportion of palm oil has been used as a biofuel, mostly in the transport sector as biodiesel derived from methyl esters of the oil. Most palm biodiesel is consumed locally in Malaysia and Indonesia. This is due to government-supported mandates that enforce the mixing of palm biodiesel with petroleum-derived diesel. However, the use of palm biodiesel as a carbon–neutral fuel in the wider global transport sector has proved to be controversial, especially in the EU (Muzii 2019). Until very recently, a substantial and growing amount of palm biodiesel, totalling 4.9 Mt in 2018, was used in the EU. As shown in Fig. 6, for over a decade the EU has steadily increased its imports of palm oil for fuel use while the amount used for food, feed and oleochemicals has declined from a high of almost 4 Mt to about 2.7 Mt (Chandran 2019). These data show that in 2018 the EU imported a total of 7.6 Mt palm oil but only 2.7 Mt (36%) of this was for food and personal care use, while the remaining 4.9 Mt (64%) was for use as transport biodiesel or fuel oil (e.g. for electricity generation).

As described above, concerns about the environmental impact of oil palm cultivation and the use of food crops for biofuel, coupled with recent advances in electric vehicle (EV) technologies, mean that the EU is now moving away decisively from both crop-based biofuels and fossil fuels, with many countries seeking to replace all carbon-based fuels by 2050. In the medium term, as fossil oil use continues to decline and its price remains low, there are few prospects that palm biodiesel will compete effectively on price in international markets. This is likely to reduce the global market for palm biodiesel, although the additional palm oil that this would release should still be in high demand for edible uses. For example, Corley and Tinker 2015) estimated that, by 2050, a further 6 Mha of land could be required to meet total oil palm production requirements, which is a formidable challenge in view of the scarcity of environmentally suitable land. However, if most of the current palm that is diverted to biodiesel is switched to food use, about 3–4 Mha of this additional land would not be required.

### Production issues

**Fig. 7:**
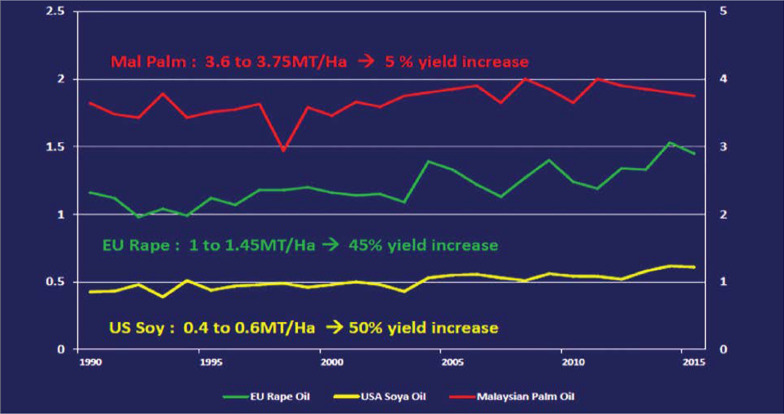
Stagnation of average oil yield (in tonnes per hectare) in Malaysian OP crops (right-hand axis) compared to two major competitor oilseeds, rapeseed and soybean (left-hand axis). Source: Ref. Chandran (2019)

On the production or supply side, the oil palm sector faces several significant challenges that include new scientific advances, changing patterns of global trade and consumer sentiment, and the related issues of labour and mechanization. The efficiency and effectiveness of plantation management varies greatly across the sector, both among large commercial enterprises and individual smallholders. One of the most remarkable features of the oil palm is the stagnation in yields at values around or under 4 t/ha over the past two decades (Chandran 2019). As shown in Fig. 7, this is in marked contrast to other major crops, including oilseeds, which have shown consistent yield increases in response to factors such as biological improvements, improved management and more efficient transport and supply chain infrastructure. In some cases, modelling analysis can provide new insights into plantation management that suggest possible improvements. A recent example is the application of model optimization and heuristic techniques that indicated significant potential for yield improvements by reducing the harvest cycle length from 19.6 to 8.3 days in a plantation in Columbia (Escallón-Barrios et al. 2020). Innovative new ideas for ‘smart’ oil palm mills have also been advanced (Isaac 2019) as well as the use of digital technologies, such as blockchain, to enhance the performance and transparency of supply chains (Keong 2019).

The oil palm cropping system is unusual in its continued reliance on large amounts of relatively unskilled manual labour that must operate in a humid and hot tropical climate on a year-round basis (Crowley 2020). During recent decades many plantations have increasingly relied on temporary migrant labour, but low wages and increasing incomes from alternative forms of employment have created staff shortages, which were greatly exacerbated by the COVID-19 pandemic (Crowley 2020; Raghu 2021). These problems have been compounded by allegations of poor labour practices in some plantations that led to the blacklisting of some of the largest commercial companies and import bans by the US Customs and Border Protection in 2020–21 (Jamal 2021).

In the long term, the most realistic solution to the current labour problems that plague the sector is to introduce more mechanization and shorter plants, as has been done for several other staple monocot and tree crops (Murphy 2011). One way of facilitating mechanization and increasing yield is to use modern molecular breeding approaches to modify crop architecture, for example to reduce trunk height as has been done with apples and major cereals such as wheat and rice (Murphy 2011; Nagai et al. 2020). Interestingly, a very recent study has identified three major QTLs associated with oil palm height on chromosome 11, which could facilitate the breeding of shorter and more compact palms for enhanced yield and ease of harvesting (Teh et al. 2020). Replanting of ageing and/or poorly performing palms is a vitally important strategy for improving the yield, and hence the overall sustainability and environmental footprint of oil palm crops. This applies to both large commercial growers and smallholders, many of whom use inferior seeds bought from middlemen with no record of their provenance. While there have been government initiatives in Malaysia and Indonesia, these efforts need to be redoubled and made more effective (Shehu et al. 2020; Yahya et al. 2020; Oosterveer 2020).

### Sustainability and environmental challenges

The use of oil palm as a food ingredient in the large EU market has been in steady decline over the past decade (Fig. 6). There is little doubt that part of this decline has been due to adverse consumer sentiment about the oil palm industry in general and there are now discussions in the EU to require verifiable ‘point of origin’ declarations for all food-grade palm oil (Southey 2020). This could mean that any oil that cannot be reliably identified as from a sustainably certified source, such as RSPO, might not be imported into the EU. Clearly the industry needs to address these certification and authenticity issues in its supply chains to ensure that it becomes fully compliant with the requirements of its second largest customer, namely the EU.

## Conclusions

The global oil palm industry is a major component of contemporary agriculture, supplying food to billions of people, plus a host of non-food products that include strategically vital cleaning products used in critical health care settings. However, there are well founded concerns about the expansion of oil palm plantations into sensitive habitats, such as highly biodiverse tropical forests and peatlands (Meijaard and Sheil 2019; Meijaard et al. 2018). There are no viable alternatives to oil palm in terms of its yield and delivery of a range of specific oils for human use (Parsons et al. 2020). It is therefore important to implement transparent and effective certification schemes right across the industry to guarantee that oil palm products can be labelled as being derived from environmentally sustainable and socially responsible sources. It is also important to recall that deforestation and habitat loss are also associated with the second most important global oil crop, soybean. Policymakers may therefore need to consider ways to reduce the demand for oils more specifically and for unhealthy ultra-processed foods more broadly. The industry also needs to redouble its efforts to engage with global consumers in a constructive dialogue aimed at addressing its image problem and explaining the many benefits of its products (Reardon et al. 2019; Borrello et al. 2020). Oil palm crops face many other challenges, including emerging threats from climate change and the likelihood of new pests and diseases, that require more effective international collaboration. The influential players in the industry need to interact with the key organizations and countries now fully committed to reducing climate change. Nevertheless, new breeding technologies are providing the promise of improvements in some areas, such as much higher yielding varieties, improved oil profiles, enhanced disease resistance and modified crop architecture to enable mechanization of fruit harvesting.

## Data Availability

Not applicable.

## Abbreviations

BSR: Basal stem rot
FFB: Fresh fruit bunches
GHG: Greenhouse gas
GM: Genetic manipulation (via transgenesis)
GWAS: Genome-wide association studies
IP: Identity preserved
LCA: Life cycle assessment (or analysis)
Mt: Million tonnes
ha: Hectare
OP: Oil palm
QTL: Quantitative trait locus
RSPO: Roundtable on sustainable palm oil
t: Tonne
